# Battlefield acupuncture for chronic musculoskeletal pain in cancer survivors: a novel care delivery model for oncology acupuncture

**DOI:** 10.3389/fpain.2023.1279420

**Published:** 2023-12-05

**Authors:** Yi Lily Zhang, Jun J. Mao, Q. Susan Li, Matthew Weitzman, Kevin T. Liou

**Affiliations:** Integrative Medicine Service, Department of Medicine, Memorial Sloan Kettering Cancer Center, New York, NY, United States

**Keywords:** cancer, pain, battlefield acupuncture, auricular acupuncture, clinical trial, care delivery

## Abstract

**Introduction:**

Battlefield Acupuncture (BFA), a standardized auricular acupuncture protocol, is widely used for pain in the military but is not well-studied in oncology. This study examined cancer survivors who received BFA for pain.

**Methods:**

This is a secondary analysis of a randomized trial that compared the effectiveness of BFA and electroacupuncture vs. usual care for chronic musculoskeletal pain in cancer survivors. This study focused on participants randomized to BFA. Participants received 10 weekly treatments. Needles were placed until one of these stop conditions were satisfied: ten needles were administered; pain severity decreased to ≤1 out of 10; patient declined further needling, or vasovagal reaction was observed. Pain severity was assessed using Brief Pain Inventory. Responders were those with ≥30% pain severity reduction. We examined pain location, BFA stop reason, and pain reduction of participants during the first session. We also examined which factors predicted responder status after the first session (week 1) or the full treatment (week 12).

**Results:**

Among 143 randomized to BFA, most common pain locations were lower back (30.8%) and knee/leg (18.2%). Of 138 who initiated treatment, 41 (30.0%) received ten needles; 81 (59.1%) achieved pain ≤1; 14 (10.2%) declined further needling; and 1 (0.7%) had vasovagal reaction. BFA reduced pain severity by 2.9 points (95% CI 2.6 to 3.2) after the first session (*P* < 0.001). After adjusting for baseline pain severity, responders at week 1 were 2.5 times more likely to be responders at week 12, compared to those who were non-responders at week 1 (AOR 2.5, 95% CI 1.02 to 6.11, *P* = 0.04). Among those who achieved pain ≤1, 74% were responders at week 12, a higher proportion compared to the proportion of responders among those who received ten needles (39.5%), those who declined further needling (50%), and those with vasovagal reaction (0.0%) (*P* = 0.001). Those with pain in proximal joints had a higher proportion of responders at week 12, compared to those with pain in distal joints (64.2% vs. 20%, *P* = 0.008).

**Conclusion:**

Specific factors may predict the likelihood of achieving meaningful pain reduction from BFA. Understanding these predictors could inform precision pain management and acupuncture delivery models.

## Introduction

Pain is one of the most common and challenging symptoms to treat in the cancer population (1–3). While opioids and other analgesic medications are cornerstones of cancer pain management, opioid use may be associated with adverse outcomes in cancer populations (4, 5). There is also a growing concern about polypharmacy in oncology (6, 7), highlighting the need for non-pharmacological pain treatment options. In 2022, the American Society of Clinical Oncology (ASCO) and the Society for Integrative Oncology (SIO) published a joint guideline recommending the use of acupuncture for cancer pain management (8). However, patient-, provider-, and system-related factors may present barriers to the integration of acupuncture into oncology, highlighting the need for novel care delivery models (9).

Battlefield Acupuncture (BFA) is a standardized auricular acupuncture protocol developed by Dr. Richard Niemtzow and implemented within the Veterans Health Administration to address acute and chronic pain conditions in the United States military population (10, 11). Retrospective studies of BFA have suggested benefits for pain (12, 13), but rigorous randomized controlled trials of this acupuncture modality have not been conducted in cancer populations (14).

Our group previously published the primary findings of the PEACE trial, a randomized controlled trial that compared electroacupuncture and BFA to usual care for chronic musculoskeletal pain in cancer survivors (15). The parent trial found that a 10-week treatment course of BFA or electroacupuncture produced significantly greater and durable pain reduction over 24 weeks relative to usual care; however, the non-inferiority of BFA to electroacupuncture was not demonstrated (15). In this current study, we conducted a secondary analysis of the PEACE trial to examine the clinical characteristics, acupuncture procedure details, and treatment responses of cancer survivors who received BFA. The goal of the current study is to better understand the role of BFA in cancer pain management and help inform novel acupuncture care delivery models.

## Method

### Study design, participants, and procedures

This study is a secondary analysis of a 3-arm, parallel-group, single-center, multi-site randomized controlled trial investigating the comparative effectiveness of BFA or electroacupuncture vs. usual care for chronic musculoskeletal pain in cancer survivors (15). The original study protocol was described previously (16), and the primary findings have been published (15). In brief, the parent study was conducted from March 2017 to April 2020. Eligible participants were survivors with a prior cancer diagnosis and no current evidence of disease who reported musculoskeletal pain with a duration of at least 3 months and a worst pain severity in the past week of ≥4 on a 0–10 numerical rating scale (0 = no pain, 10 = worst pain imaginable). Participants were randomized in a 2:2:1 ratio to electroacupuncture, BFA, or usual care. Interventions were delivered weekly over 10 weeks. The primary outcome was the change in the average pain severity score on the Brief Pain Inventory (BPI) from baseline to week 12. Participants were followed for a total duration of 24 weeks. The study was approved by the institutional review board at Memorial Sloan Kettering Cancer Center (IRB No: 16–157) and conducted in accordance to the guidelines from the Consolidated Standards of Reporting Trials (CONSORT) (17), the Standard for Reporting Interventions in Clinical Trials of Acupuncture (STRICTA) (18), and the Declaration of Helsinki. The current study focused on the participants randomized to BFA; participants randomized to electroacupuncture or usual care were excluded from the analyses.

### Battlefield acupuncture intervention

Licensed acupuncturists followed the standardized protocol developed by Dr. Richard Niemtzow (10, 11). Unlike conventional forms of acupuncture, BFA was not customized based on pain location or co-morbid symptom presentation. Consistent with the original BFA protocol, acupuncturists used ASP needles (Sedatelec), rather than conventional acupuncture needles. ASP needles were small (<3 mm), dart-shaped, semi-permanent metallic needles applied with a plastic injector. Patients' ears were sterilized prior to the BFA procedure. The acupuncturist placed an ASP needle in the cingulate gyrus point on one ear, then instructed patients to walk for 1 min. Afterwards, the acupuncturist asked patients to rate their pain severity. If severity remained greater than 1 out of 10, the acupuncturist placed a needle in the cingulate gyrus point of the other ear. This process was repeated for each remaining ear point: thalamus, omega 2, point zero, and shen men (Supplementary Materials). The acupuncturist stopped placing needles if any of the stop conditions were satisfied: (1) the maximum of 10 needles were administered; (2) pain severity decreased to 1 or 0 out of 10, (3) patient declined further needling; or (4) vasovagal reaction was observed. The total treatment duration was approximately 10 to 20 min, depending on how many needles were administered. ASP needles remained in place after the treatment. Patients were instructed on how to safely remove the ASP needles after 3 or 4 days.

### Outcomes

The average pain severity score was calculated from the BPI at baseline and week 12. Before each BFA treatment session, patients were also asked to rate their current pain severity on a 0–10 numerical rating scale (0 = no pain, 10 = worst imaginable pain). After each treatment session, patients were asked to rate their pain again using the same scale. Pain responders were defined as BFA recipients who achieved ≥30% reduction in pain severity.

After each session, acupuncturists documented BFA procedure details, including number of ASP needles used and the stop reasons: (1) the maximum of 10 needles were administered; (2) pain severity decreased to 1 or 0 out of 10, (3) patient declined further needling; or (4) vasovagal reaction was observed. The National Cancer Institute's Criteria for Adverse Events (CTCAE), version 5 was used to classify each adverse event (AE) (19). These outcomes were documented in the Research Electronic Data Capture (REDCap) data management platform (20).

### Statistical analyses

The demographic and clinical characteristics of participants were presented using descriptive statistics. Acupuncture procedure details and adverse events data were extracted from REDCap and summarized descriptively. A paired *t*-test was used to evaluate the pre-post change in pain severity. Chi square analyses were used to evaluate whether proportion of week 12 responders differed by specific characteristics, such as pain body location or BFA stop reason. Logistic regression was used to evaluate predictors of week 1 or week 12 responder status after adjusting for baseline pain severity. Statistical analyses were performed using STATA software (Windows version 15.0, Stat Corp LP, College Station, TX). All analyses were two-sided with a *P* < 0.05 indicating statistical significance.

## Results

**Figure 1:**
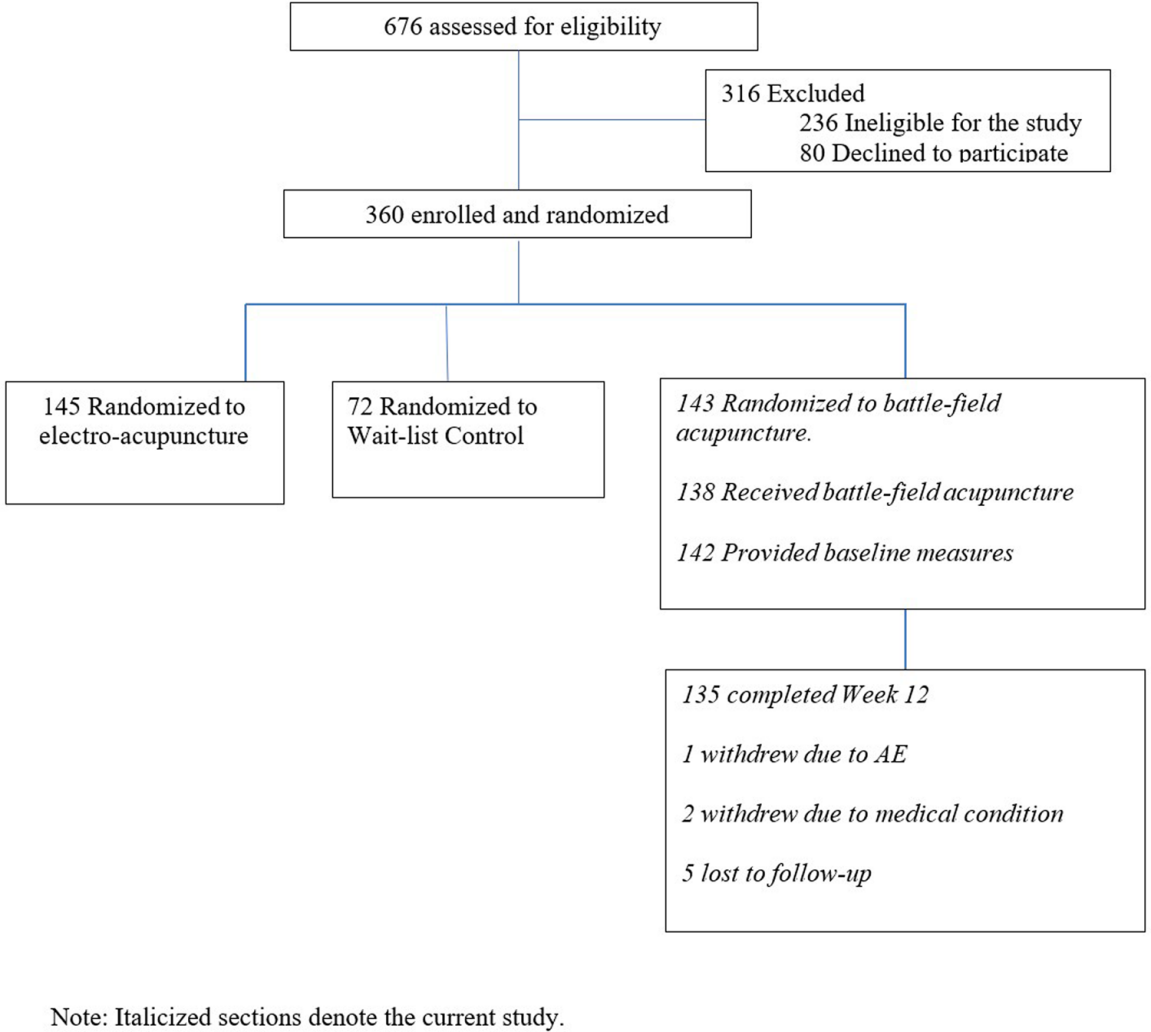
CONSORT diagram.

In the parent trial, 676 patients were screened, and 316 were excluded due to study ineligibility, lack of interest, or issues related to scheduling or transportation. The remaining participants were randomized to BFA (*N* = 143), electroacupuncture (*N* = 145), or usual care (*N* = 72). The current study includes 143 participants randomized to BFA (Figure 1). Socio-demographics and clinical characteristics of BFA recipients can be found in Table 1. The mean (SD) age was 62.6 (11.3) years, 65.7% were female, and 23.8% were non-white (*N* = 17 Black participants, *N* = 11 Asian, and *N* = 6 reporting more than once race). The most common pain locations were lower back (30.8%) and knee/leg (18.2%).

**Table 1:** Socio-demographic and clinical characteristics of BFA recipients.

Characteristic	*N*	%
Age—year	62.6 ± 11.3	
Female sex—no. (%)	94 (65.7)	
Race—no. (%)		
– White	109	76.2
– Non-white	34	23.8
Ethnicity—no. (%)		
– Hispanic	12	8.5
– Non-Hispanic	130	91.5
Cancer Type—no. (%)		
– Breast	67	46.9
– Prostate	18	12.6
– Colorectal/Gastrointestinal	4	2.8
– Lymphoma	19	13.3
– Melanoma	8	5.6
– Lung	7	4.9
– Other	20	14
Cancer Treatments—no. (%)		
– Surgery	103	72
– Chemotherapy	56	39.2
– Radiation	78	54.6
– Immuno/Biological	12	8.4
– Hormonal	31	21.7
Years since Cancer Diagnosis—year	6.1 ± 6.8	
Duration of Pain Symptom—year	4.7 ± 6.3	
Location of Pain—no.(%)		
– Neck	15	10.5
– Upper back	7	4.9
– Lower back	44	30.8
– Shoulder/Arm/Elbow	24	16.8
– Wrist/Hand	5	3.5
– Hip/Thigh	17	11.9
– Knee/Leg	26	18.2
– Ankle/Feet	5	3.5
Baseline measures		
Brief Pain Inventory Severity (Mean/SD)		
– Severity score	5.0 ± 1.7	
– Worst pain item	6.6 ± 1.8	
– Average pain item	5.4 ± 1.7	
Brief Pain Inventory Interference (Mean/SD)	4.7 ± 2.2	

Of the 138 participants who underwent BFA treatment, 41 (30.0%) received a maximum of 10 needles in the first session; 81 (59.1%) stopped treatment prior to receiving 10 needles because their pain severity was reduced to 1 or less; 14 (10.2%) declined further needling before the 10 needles were administered; and 1 (0.7%) stopped treatment before receiving 10 needles because a vasovagal response was observed.

These 138 BFA recipients reported a mean (SD) pain severity of 4.6 (1.9) before the first BFA treatment and a mean (SD) pain severity of 1.7 (2.0) after the first treatment, which translates to a pain reduction of 2.9 (95% CI 2.6 to 3.2, *P* < 0.001). After adjusting for baseline pain severity, BFA recipients who were pain responders after the first session were 2.5 times more likely to be pain responders at week 12, compared to those who were non-responders after the first session (AOR 2.5, 95% CI 1.02 to 6.11, *P* = 0.044).

**Figure 2:**
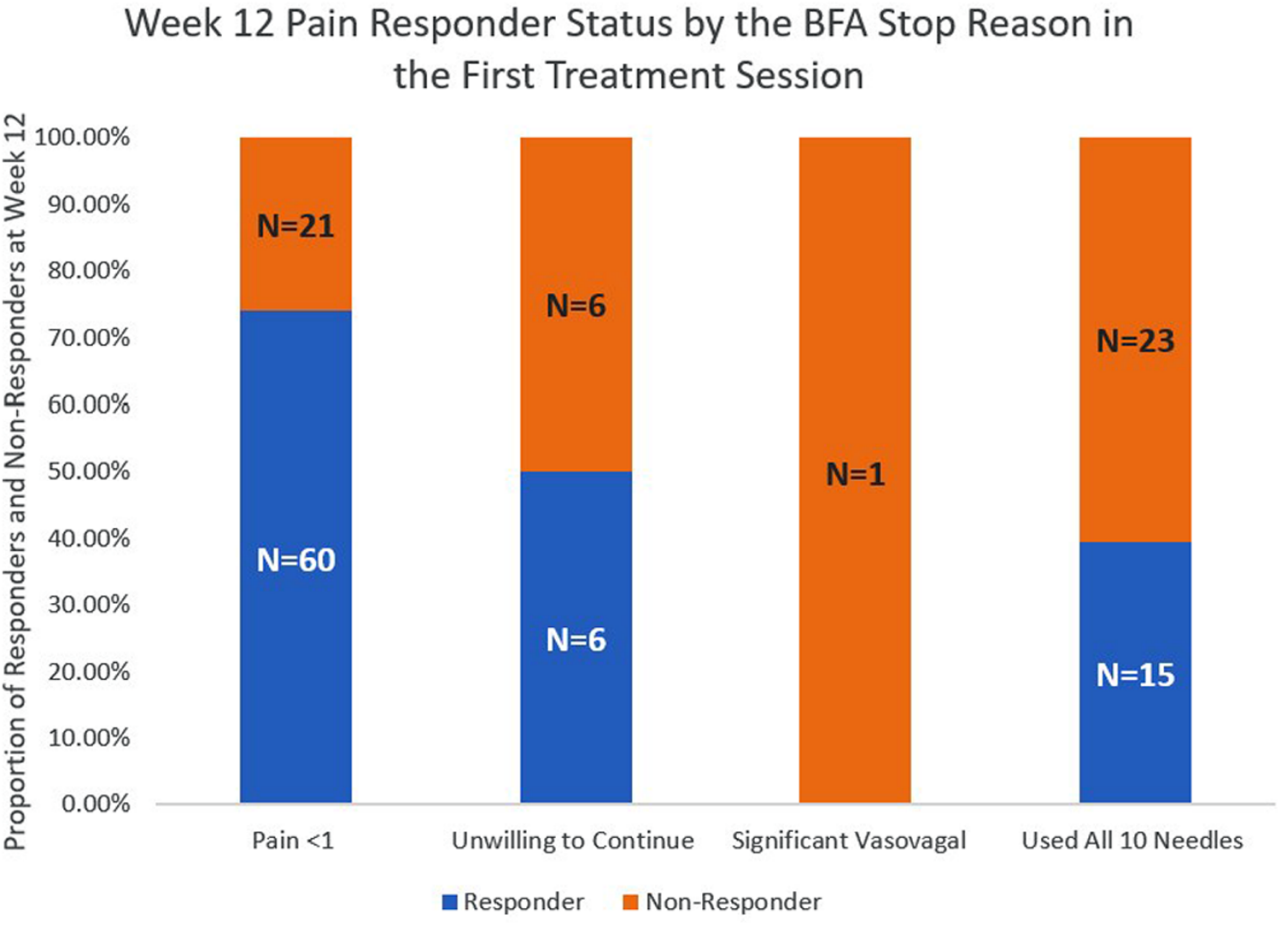
Week 12 pain responder Status by the BFA stop reason in the first treatment session.

**Figure 3:**
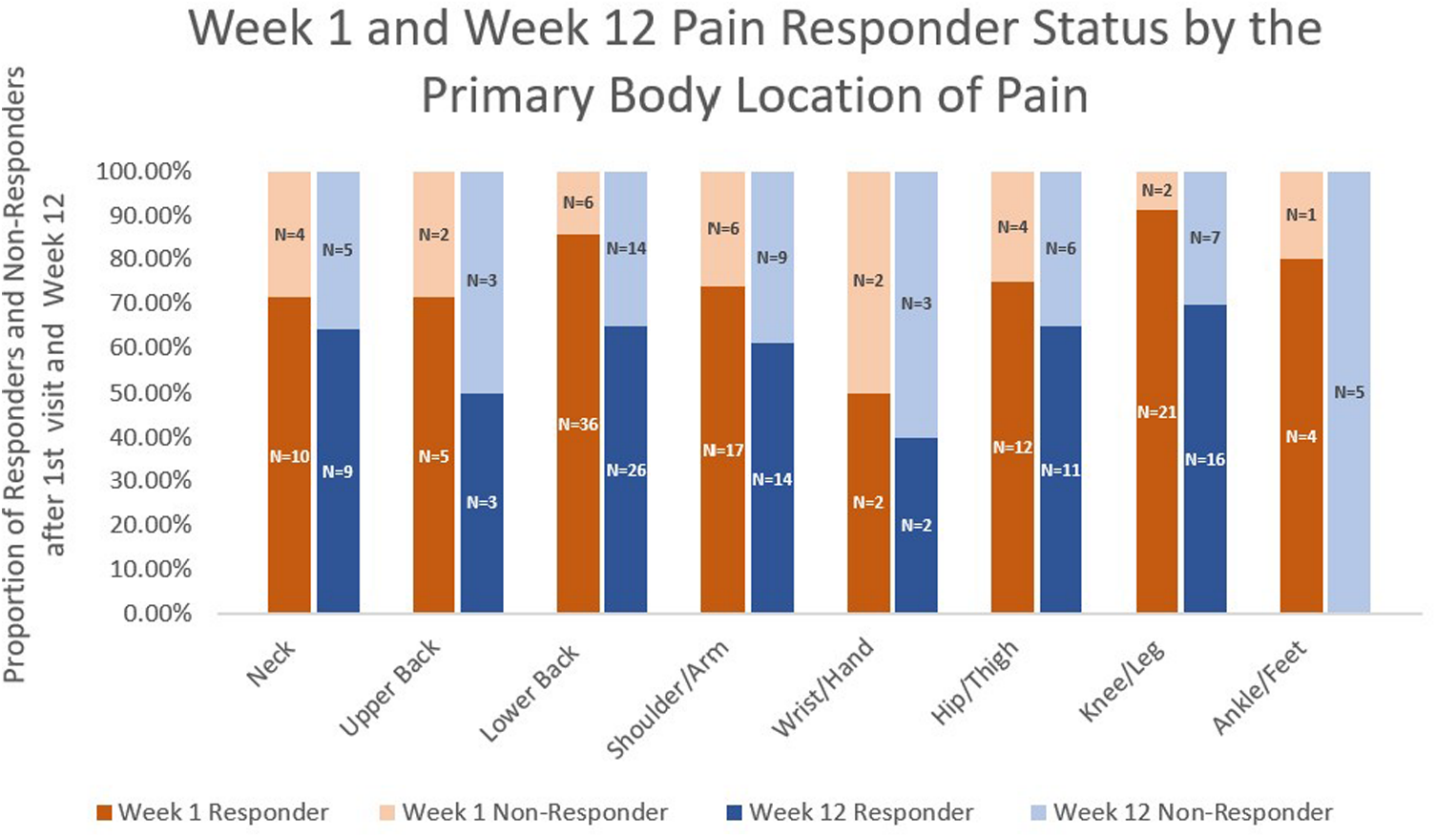
Week 1 and week 12 pain responder status by the primary body location of pain.

The pain responder status at week 12 also differed by stop reason (Figure 2) and by pain location (Figure 3). Regarding stop reasons, the proportion of pain responders at week 12 were highest among those whose pain severity was reduced to 1 or less prior to receiving 10 needles in the first session (74.1%), followed by those who declined further needling (50%), those who received a maximum of 10 needles (39.5%), and the participant who experienced vasovagal reaction (0.0%) in the first session (*P* = 0.001). Regarding pain location, the highest proportion of pain responders at week 12 were observed among participants with knee/leg pain (69.6%), lower back pain (65.0%), hip/thigh pain (64.7%), neck pain (64.3%), shoulder/arm pain (60.9%), upper back pain (50%), and the lowest proportion were observed among participants with wrist/hand (40%) and ankle/feet (0%). The proportion of pain responders at week 12 differed between those whose pain was located proximally (neck, lower back, upper back, hip/thigh, shoulder/arm; knee/leg) vs. distally (wrist/hand, ankle/foot); 64.2% of BFA recipients with pain in more proximal joints were responders at week 12, compared to 20.0% of those with pain in more distal joints (*P* = 0.014).

With regards to AEs, 4 BFA recipients experienced grade 1 dizziness after the first treatment session.

## Discussion

In this secondary analysis of a randomized controlled trial, we found that clinical responses to the first BFA session could potentially help predict which patients have a high likelihood of experiencing clinically meaningful pain reduction after undergoing a full treatment course. We also found that pain response rates may differ by primary pain location. Although preliminary, these findings can guide future research to determine the optimal role of BFA in cancer pain management.

Our study contributes to the emerging literature on the use of BFA for pain management in various patient populations. Compared to other BFA research, most of which was conducted in military populations, the cancer survivors in our study reported lower baseline pain severity but similar magnitude of pain reduction after the first treatment with BFA. One study (*N* = 284 veterans) found that the mean pain severity decreased from 6.8 to 4.5 after the first BFA treatment, a reduction of 2.3 points (12). In another cross-sectional study of over 11,000 veterans, mean pain severity decreased from 6.3 to 3.8 after the first BFA treatment, a reduction of 2.5 points (13). These findings suggest that BFA may provide fast-acting pain relief for some patients after a single session; however, BFA may not be appropriate for everyone. Our findings suggest that approximately one in ten BFA recipients declined further needling due to difficulties tolerating ear discomfort from the needles. Also, because the BFA protocol involves patients ambulating after each needle is administered, patients with dizziness or other mobility issues may not feel comfortable with this procedure. In addition to pain outcomes, future studies on BFA should systematically document AEs to investigate the safety and appropriate delivery of this treatment modality.

Our study also suggests that the BFA stop reasons in the first treatment session could be a useful predictor of pain response at the end of the full treatment course; however, further research is needed to confirm these preliminary findings. Unfortunately, prior studies have limited information regarding the stop reasons related to the BFA procedure. In the previously described study by Zeliadt et al., only one out of the 57 clinics recorded information about treatment complications; of the 1,946 BFA treatments delivered at the clinics, 12 (0.6%) had documentation indicating that the patient requested the treatments be stopped, although the exact stop reasons were not provided (13). Future BFA studies should carefully record and publish stop reasons to facilitate more rigorous research in this area.

Interestingly, we found that treatment response rates may differ by the primary pain location. Patients with pain in proximal joints appear to respond better to BFA relative to those with pain in distal joints; however, these findings need to be confirmed in a larger study with adequate representation across the various sub-groups for each pain location. Prior studies of BFA and other forms of acupuncture have examined treatment responses primarily through the lens of general pain conditions (e.g., osteoarthritis) (21) or medical populations (e.g., cancer) (14), rather than specific pain locations. However, based on our study, examining whether treatment responses differ by distinct anatomical pain locations may also be a worthwhile avenue for research.

Our findings have several potential implications for clinical practice and research related to oncology acupuncture. In settings where conventional forms of acupuncture are not widely available, patients with pain in proximal joints could be triaged to BFA due to higher likelihood of response to BFA, whereas those with distal joints could be prioritized for conventional acupuncture due to lower likelihood of response to BFA. Alternatively, patients could undergo an initial BFA treatment to guide clinical decision-making; those who achieve pain reduction to ≤1 in the first session could continue with BFA due to higher likelihood of response, whereas those who received the maximum of ten needles (or had other stop reasons) could be referred to receive conventional acupuncture due to lower likelihood of response to BFA. These types of clinical scenarios should be further investigated using appropriate study designs, such as adaptive trials. If confirmed in rigorous trials, our findings can be leveraged to guide allocation of healthcare resources and develop novel care delivery models for acupuncture and other non-pharmacological treatments.

This exploratory study should be interpreted in the context of several limitations. First, this study focused primarily on data from the first BFA session to identify potential predictors of treatment response. Future studies should examine whether data from the subsequent BFA sessions can yield useful information about predictors of treatment response. Second, some of the sub-groups examined in this study had low numbers of participants (e.g., patients with ankle/foot pain and/or with vasovagal reaction). Larger studies with adequate representation across sub-groups will be needed to confirm our findings. Third, pain was examined as an isolated symptom in this study, even though it frequently co-occurs with multiple symptoms. Future studies should examine the effects of BFA on other symptoms co-morbid with pain, as well as the potential role of co-morbid symptoms in predicting treatment response. Fourth, while pain medications were tracked at several timepoints during the parent trial, we did not collect data on all types of pain treatments used by participants on the day of the BFA sessions; thus, concurrent use of other pain treatments could have potentially affected the reporting of pain severity before and after BFA sessions. Finally, this is a secondary analysis of a randomized controlled trial. Therefore, the findings are exploratory and intended to be hypothesis-generating.

Despite these limitations, this study examined data from the first and largest randomized controlled trial of BFA conducted in an oncology setting. Our preliminary findings suggest that pain location and responses to the first BFA session may have clinical utility as a predictor of pain responses after a full treatment course of BFA. Better understanding of these potential predictors of BFA response can lead to the development of precision pain management approaches and novel acupuncture delivery models in oncology.

## Data Availability

The raw data supporting the conclusions of this article will be made available by the authors, without undue reservation.
