# Supplementary figures and images for: Battlefield acupuncture for chronic musculoskeletal pain in cancer survivors: a novel care delivery model for oncology acupuncture
Source: Front Pain Res (Lausanne). 2023 Dec 5;4:1279420. doi: 10.3389/fpain.2023.1279420 (PMC10728598; doi:10.3389/fpain.2023.1279420)

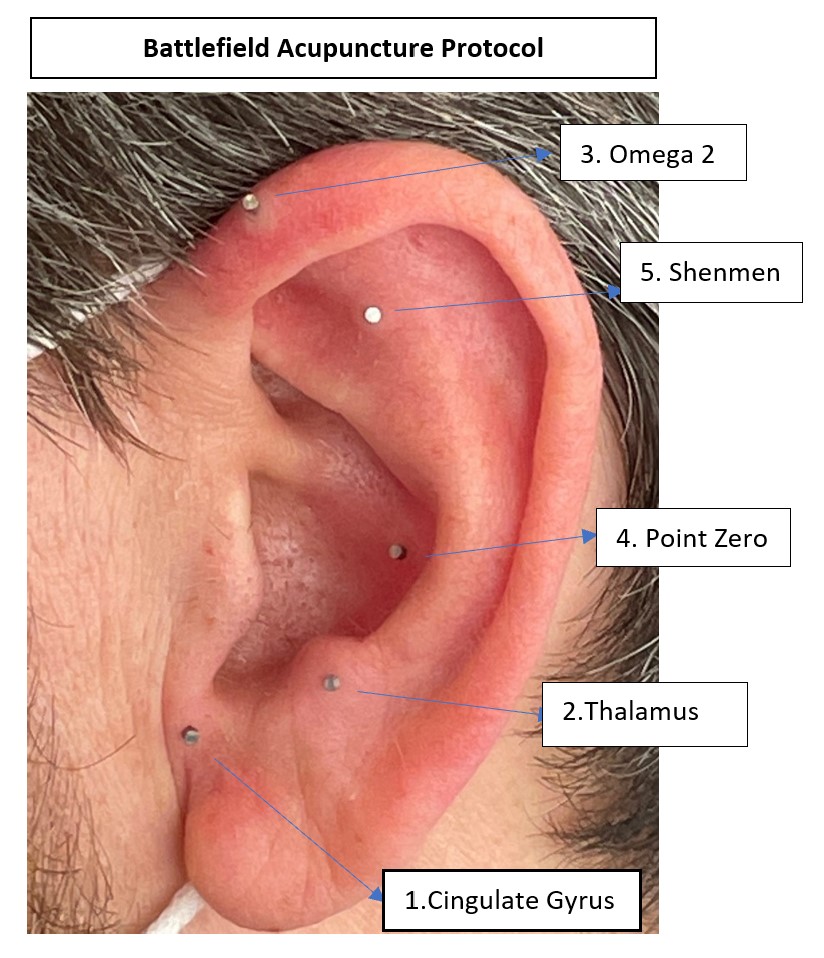

Supplement: Supplementary file 1 [file Image1.jpeg]
